# Nanosized Hydroxyapatite Coating on PEEK Implants Enhances Early Bone Formation: A Histological and Three-Dimensional Investigation in Rabbit Bone

**DOI:** 10.3390/ma8073815

**Published:** 2015-06-25

**Authors:** Pär Johansson, Ryo Jimbo, Yusuke Kozai, Takashi Sakurai, Per Kjellin, Fredrik Currie, Ann Wennerberg

**Affiliations:** 1 Department of Prostodontics, Faculty of Odontology, Malmö University, Malmö S-214 21, Sweden; E-Mails: ryo.jimbo@mah.se (R.J.); ann.wennerberg@mah.se (A.W.); 2 Department of Radiopraxis Science, Kanagawa Dental University, Yokosuka, Kanagawa Prefecture 238-8580, Japan; E-Mails: kozai@kdu.ac.jp (Y.K.); sakurait@kdcnet.ac.jp (T.S.); 3 Promimic AB, Stena Center 1B, Göteborg S-412 92, Sweden; E-Mails: per@promimic.se (P.K.); fredrik@promimic.se (F.C.)

**Keywords:** polyether ether ketone, HA, biomaterial, histology

## Abstract

Polyether ether ketone (PEEK) has been frequently used in spinal surgery with good clinical results. The material has a low elastic modulus and is radiolucent. However, in oral implantology PEEK has displayed inferior ability to osseointegrate compared to titanium materials. One idea to reinforce PEEK would be to coat it with hydroxyapatite (HA), a ceramic material of good biocompatibility. In the present study we analyzed HA-coated PEEK tibial implants via histology and radiography when following up at 3 and 12 weeks. Of the 48 implants, 24 were HA-coated PEEK screws (test) and another 24 implants served as uncoated PEEK controls. HA-coated PEEK implants were always osseointegrated. The total bone area (BA) was higher for test compared to control implants at 3 (*p* < 0.05) and 12 weeks (*p* < 0.05). Mean bone implant contact (BIC) percentage was significantly higher (*p* = 0.024) for the test compared to control implants at 3 weeks and higher without statistical significance at 12 weeks. The effect of HA-coating was concluded to be significant with respect to early bone formation, and HA-coated PEEK implants may represent a good material to serve as bone anchored clinical devices.

## 1. Introduction

The search for the optimal replacement for human bone began with metallic devices in orthopedic, dental and trauma applications [1,2,3]. However, concern was raised regarding the release of toxic elements such as nickel and chromium due to corrosion. Furthermore a mechanical mismatch between bone and metallic implants was another concern. The stiffness of metal implants creates abnormal forces on the adjacent structures, sometimes resulting in stress shielding and segment degeneration [4,5]. Some of these unfavorable properties of metallic implants have been shown to cause osteolysis, allergenicity and implant detachment [6,7,8].

Today, the search for optimal implants continues with the ambition to compensate for adverse tissue reactions due to ionic leakage and mechanical mismatches. Polyether ether ketone (PEEK) has shown promising abilities as an alternative to metal implants due to its chemical resistance, mechanical properties and radiolucency [9]. PEEK has an elastic modulus between the value of cortical and cancellous bone, *i.e.*, considerably lower than metal (PEEK: 3.2 GPa, CP Ti: 114 GPa, cortical bone: 15–30 GPa, cancellous bone: 0.5–1.5 GPa). Furthermore, PEEK may be machined or molded into any shape and size [6,9,10,11,12,13]. Regardless of these attributes, PEEK is relatively bio-inert and hydrophobic and has been found to osseointegrate poorly in its pure form [9,14].

To increase the ability to osseointegrate, incorporation of hydroxyapatite (HA) into PEEK or onto its surface has been assessed. Although Ha-coating of PEEK has proven successful in increasing the bone incorporation, some studies have resulted in degraded mechanical abilities or reduced physical bonding between the substrate and the coated layer [15,16,17,18,19]. Deposition of a thin coating layer of HA on titanium implants has shown less degradation and dissolution [20]. In this study, PEEK implants were coated with HA using a unique procedure which creates a layer with a thickness measuring 20–40 nm. This technique has previously been evaluated on titanium implants with promising outcomes [21,22]. The hypothesis of this work was that if crystalline HA was applied to a PEEK implant surface, the bone area in the threads and the bone-to-implant contact would be significantly larger than neat PEEK.

Thus, the overall purpose of this study was to investigate the biological effects of nano-HA coating on PEEK implants as evaluated by histomorphometry and micro-computed tomography. The study was conducted in a rabbit model and evaluated at 3 and 12 weeks after implantation.

## 2. Results

### 2.1. Overall

All animals healed uneventfully except one who died of unknown causes 1 week after implant insertion. At each retrieval point, all implants were immobile with no clinical signs of inflammation on the skin or around the surgical sites.

### 2.2. Coating Stability

To reproduce the mechanical stress elicited when HA-coated and control PEEK implants were inserted in Sawbone material, we monitored the torque during insertion of the implants. Sawbone consists of a polyurethane foam with a filler, and is available in different porosities and densities, all designed to mimic cortico-cancellous bone proper. Monitoring the torque during the insertion gives an estimate on the mechanical forces on the implant surface.

**Figure 1:**
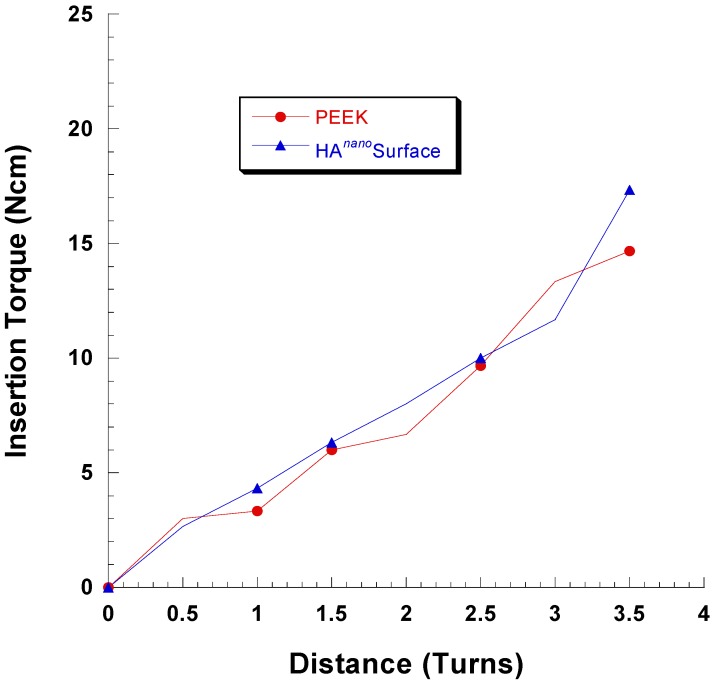
Insertion torque *vs.* distance for the coated and uncoated implants. The implant was inserted with its top at the level of the surrounding bone surface at 3.5 turns.

As seen in Figure 1, the insertion torque profiles are virtually the same for coated and uncoated implants. Therefore, even if the coated and uncoated implants have different chemistries and nanostructures, the frictional forces seem most similar for the tested implants. This finding indicates that the friction was governed by the microtopography of the implant surface.

If the coating is to work properly in a clinical situation, it is important that the majority of the coating can withstand the mechanical forces which are created during the insertion process. Measuring the adhesion strength of the coating to the underlying surface gives valuable information for predicting the durability of the coating. However, standard adhesion measurement protocols for HA coatings such as tensile testing (for example ISO 13779-4 or ASTM F1147–05) are not suitable for coatings in the nanometer regime. These protocols have been suited for plasma sprayed micrometer-thick HA-coatings only. A qualitative assessment of the adhesion strength of the HA coating can be done by performing SEM analysis of the implants prior to and after insertion and removal of the implants.

**Figure 2:**
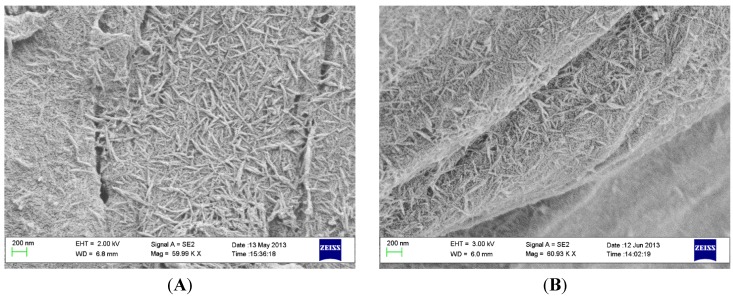
SEM images of a PEEK implant at the thread valleys (**A**) before insertion and (**B**) after insertion and removal. Scale bar = 200 nm.

As seen in Figure 2 the HA coating at the thread valleys was retained after the insertion and removal procedure. Also, along the thread “slopes,” the coating was preserved.

**Figure 3:**
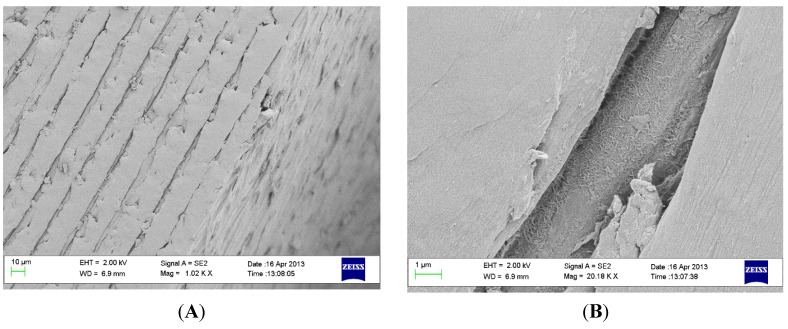
SEM images of thread edge of implant after insertion and removal. (**A**) scale bar = 10 µm and (**B**) scale bar = 1 µm.

The thread edges are generally the parts which are subjected to the highest mechanical forces during placement and removal of the implant. As seen in Figure 3, the coating is well-preserved in the small cracks and fissures on the edges, but on the flat areas which were in direct contact with the Sawbone the PEEK surface showed signs of deformation which, in turn, may lead to low surface coverage of HA crystals.

### 2.3. Micro-CT Evaluation

**Figure 4:**
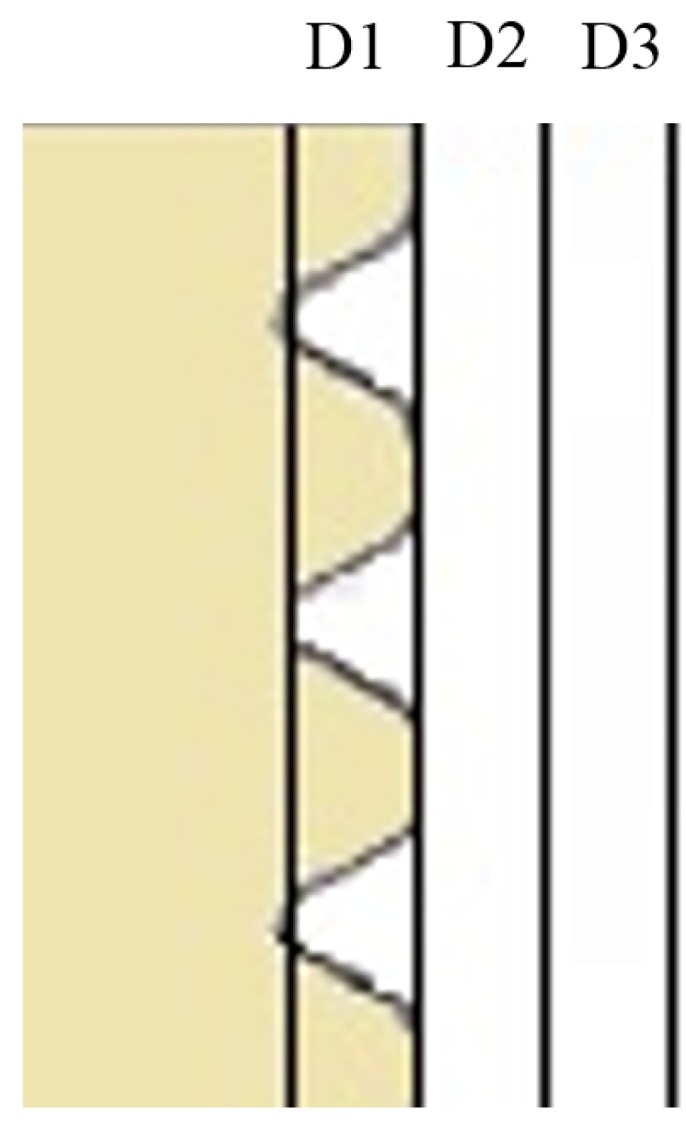
Schematic plan of the subdivision of micro-CT volume of interest (VOI). D1 is the closest to the threads and D3 is the one furthest away.

The results acquired from the micro computed tomographic evaluation showed for the full volume of interest (VOI, D1 + D2 + D3, Figure 4) no significant differences between the two groups after both 3 and 12 weeks of healing. The inner VOI (D1 closest to the implant thread) revealed, after 12 weeks only, a significant difference (*p* = 0.044) between the test and control when comparing bone surface in terms of volume (Test: 23.5% ± 1.5%, Control; 24.7% ± 4.3%) and trabecular thickness (Test: 85.5 ± 5.4 µm, Control: 82.8 ± 12.1 µm). This difference aside, there were no other significant differences between the groups or healing periods.

### 2.4. Histomorphometry

**Figure 5:**
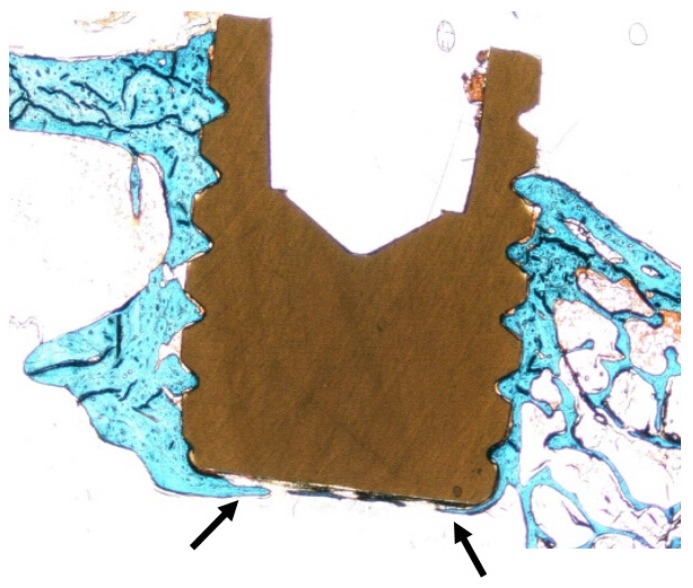
Histologic section of all threads presenting manifest bone ingrowth in all threads, indicative of osteoconductive features of the PEEK material (arrow). This actual section was from an HA-coated implant retrieved 12 weeks after surgery.

The histological sections presented homogenous blue colored staining adjacent to the implant surface of the two groups. In proximity to the surface coating there were no signs of detached coating debris or giant cells indicative of an inflammatory response. After 12 weeks most of the threads were filled with bone. Signs of osteoconductive features of HA-coated PEEK implants were observed (Figure 5).

**Figure 6:**
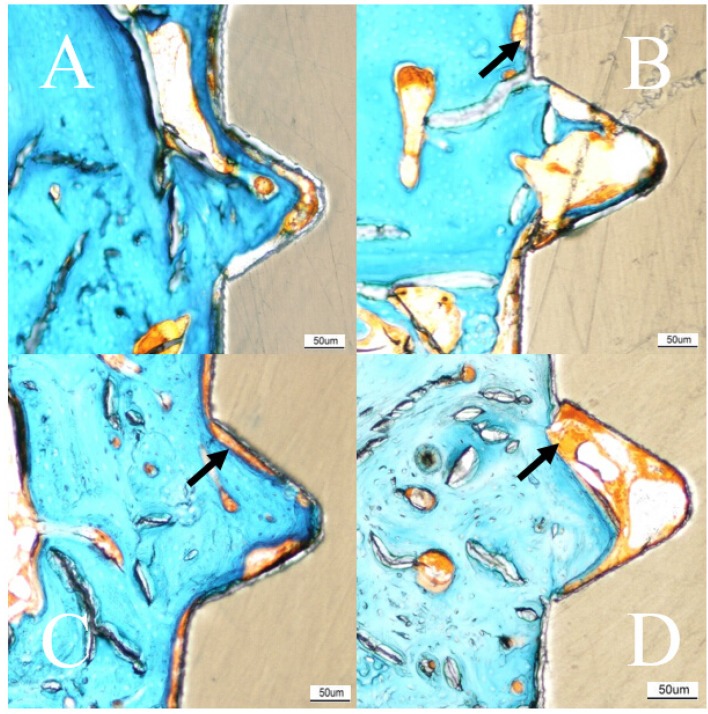
Histologic results. Masson Goldner-Trichrome stained sections of the implant after 3 and 12 weeks of healing. No soft tissue, or only sparse amounts (green), have migrated down between the implant and bone. Fibrous tissue (orange, arrow) can be viewed at the gap between the bone and implant, more commonly at the 3 week samples. (**A**) 3 weeks test (HA); (**B**) 3 weeks control; (**C**) 12 weeks test (HA); (**D**) 12 weeks control. Scale bar = 50 µm.

The histological outcomes after 3 weeks of healing revealed significantly higher BA and BIC values for test implants compared to controls (BA: *p* = 0.02, BIC: *p* = 0.024). Bone area (%, SD) after 3 weeks for test and control was 27.5 (10.6) and 17.9 (5.8) respectively and the BIC (%, SD) was test 7.94 (6.94) and control 2.91 (1.87, Figure 6A–D and Table 1).

**Table 1:** Histomorphometric results 3 and 12 weeks after implantation.

*n* = 24		Average (SD)	
		Bone-Implant Contact (BIC, %)	Bone Area (BA, %) (in Thread)
**3 weeks**	Test	7.94 (6.94)	27.49 (10.63)
	Control	2.91 (1.87)	17.91 (5.78)
	p-value	**0.016**	**0.027**
**12 weeks**	Test	6.75 (6.52)	48.72 (11.12)
	Control	4.34 (3.28)	38.27 (9.43)
	p-value	0.622	0.02

**Figure 7:**
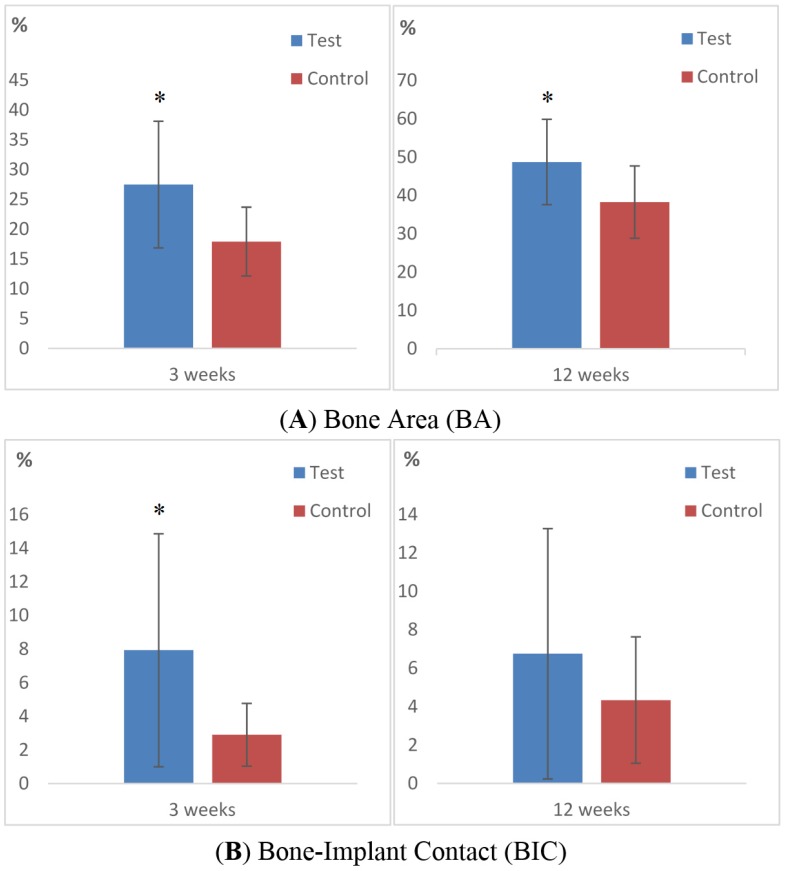
Bar graphs displaying the percentage of (**A**) bone area (BA) and (**B**) bone-to-implant contact (BIC) for test and control after 3 and 12 weeks of healing. Data are presented as mean with standard deviation. Mann Whitney U test was used for testing level of significance. (*, *p* ≤ 0.05).

The second healing point at 12 weeks showed higher BIC values for test implants compared to controls but without statistical significance. The BA (%, SD) was for test 48.7 (11.1) and control 38.3 (9.4) after 12 weeks. The BIC (%, SD) was measured to 6.75 (6.52) and 4.34 (3.28) for test and control, respectively (Figure 7).

## 3. Discussion

A nano thin hydroxyapatite coating on PEEK implants was evaluated and found to increase the bone-implant contact. This increased BIC conforms to several other studies on hydroxyapatite coated titanium [9,23].

The histologic results demonstrated bone-forming activity adjacent to the test in contrast to the control implants, since the histological BIC was significantly higher for the HA-coated PEEK implant after 3 weeks (*p* = 0.024). After 12 weeks of healing, the HA-coated implants still possessed higher BIC than the controls, but without significant difference. This trend has been confirmed in several previous studies evaluating thin coating layers [24,25,26]. Our finding with regard to a stronger bone reaction to test implants is supported by our previous biomechanical study on similar test and control implants [27]. In this previous study, the removal torque for 3 weeks was 13.0 and 7.18 Ncm, and for 12 weeks, 9.75 and 5.58 Ncm, for test and control implants, respectively. Taken together, the rate of new bone formation was enhanced for HA-coated implants in the early healing point, which also concurs with several previous reports [28,29,30]. Lee *et al.* evaluated different ceramic coatings and found that the attraction to bone is weaker with time after implantation [31]. Nonetheless, ceramic coatings differ from many metal implants, where the stability relies on the mechanical bonding created by the surface topography [32]. Having said this, with time the bone response is also weakened in metal implants [32].

The percentage of bone in contact with the uncoated control PEEK surface was surprisingly low. The BIC only increased from 2.91 percent to 4.94 percent from 3 to 12 weeks. This minor increase in BIC after 12 weeks of implantation was very different when compared to studies on machined titanium implants [33,34]. Barkamo *et al.* evaluated HA-coated and untreated PEEK implants after one healing period of six weeks. When measuring all threads they found significantly higher BIC for HA-coated PEEK but the BA was similar in both groups [35]. The finding of a BIC advantage for HA-coated PEEK was similar to this study, which also showed a significantly higher BA for HA-coated PEEK after 3 weeks. However, the absolute values cannot be compared due to the use of different drilling protocols, healing times and interpersonal perceptions of the histologic image. The removal torque results from the study of Barkamo *et al.* was comparable to our previous study [27], where HA-coated PEEK required significantly higher removal torque compared to untreated PEEK. Overall, the results from these studies are similar, and together they indicate the osteoinductive effect of an HA coating.

The surface of the test implant consisted of HA rods, nanometers in size and the overall topography was, in a previous study for both groups, classified as minimally rough (S^a^ 0.5–1.0 µm) somewhat below the optimal roughness for dental implants (S^a^ 1.5 µm) suggested by Albrektsson and Wennerberg [27,36]. Many studies have been conducted to evaluate whether nanotopography affects bone formation. Meirelles *et al.* evaluated HA-coated titanium implants with different nanotopographies in early stages of osseointegration with respect to removal torque and histologic measurements [37]. They found a correlation between chemical modification, nanotopography and increased removal torque. This study compared two groups with different chemical modification that did not change the surface topography (Test: S^a^ = 0.69 µm, control: S^a^ = 0.66 µm) [27]. Thus, only the influence of the HA coating was evaluated. The results from this and our previous study indicate, as did Meirelles *et al.*, that removal torque, BIC and BA was increased with an HA coating of the surface. The decrease of BIC after 12 weeks can be compared with the decreased removal torque, which indicates the early impact of the coating but also the disadvantage of the smooth implant surface. When it comes to evaluating bone-implant integration, the results become a mixture of several mechanisms, which are still not fully identified. For instance, the morphological appearance alone may enhance the bone-implant interlocking regardless of surface chemical modification. Therefore, this study aimed to evaluate the level of osseointegration using histology and radiology, while our previous study focused on the mechanical bond through removal torque testing [27].

The quantitative assessment of BA within the threads revealed a clear and significantly increased area for test implants (3 weeks: *p* = 0.027, 12 weeks: *p* = 0.02, Figure 6 and Figure 7). A greater amount of bone was found within the threads even newly formed bone reached the apical part of the implant. This osteoinductive feature of HA is well supported by several other studies where HA have been applied to different implant materials [27,38,39]. However, the specific osteoinductive process of ceramic coatings is still unclear but authors have reported that surface roughness and chemical composition have decisive influence on the outcome [40,41,42]. Other studies have shown that the dissolution of HA ions has an up-regulating effect on the osteoblast activity, which may explain the *in vivo* results of the present paper [43]. The precise biological events remain unclear and are in need of further study. PEEK has a radiolucent feature that gives the surgeon an improved radiographic interpretation after spinal surgery [44]. Correspondingly, in experimental setups this feature gives the researcher many advantages. The evaluation can be performed in a non-invasive manner with an intact sample, and the bone-implant interface can be assessed three-dimensionally. Therefore, in this study we chose to perform a computed tomographic evaluation before the samples were cut and ground in order to obtain a detailed view of the interface. Micro-CT is a reliable non-invasive analytical instrument to quantify the amount of mineralized bone around artificial implants and characterize the bone structures [45,46]. Together with the histological sections, a comprehensive image of the bone can be surveyed and provide a representative description of the healing event. In this report, the micro-CT evaluation was focused on potential differences in mineralized bone volume and trabecular organization. BV/TV indicates the fraction of bone in the given VOI, which explains the effect of the bioactive coating. However, the present study revealed no differences between the groups. This could be explained by an inappropriate selection of VOI which makes the small bone changes undetectable, in addition to the voxel resolution of the radiographic equipment that could have limited the possibility to detect minor deviations.

## 4. Experimental Section

### 4.1. Implant Manufacturing

**Figure 8:**
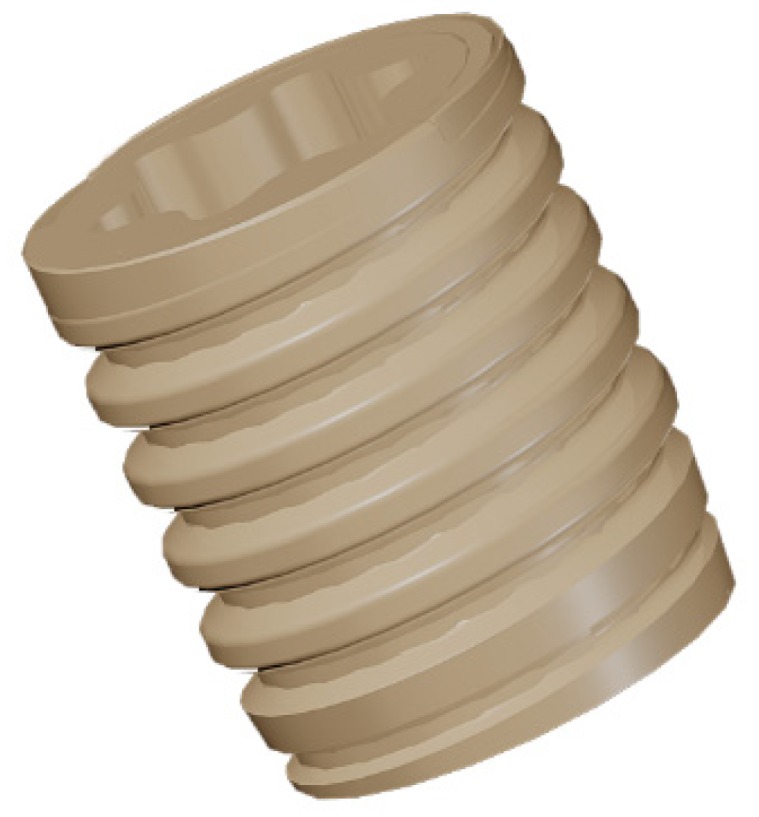
3D rendered image of the PEEK implant.

Commercial PEEK substrates (Invibio Ltd. Lancashire, UK) with non-cutting screw shape and diameter of 3.5 mm and length of 4 mm were prepared for the study by Elos Pinol A/S, Görlöse, Denmark (Figure 8).

### 4.2. Coating Preparation

Altogether, 24 implants were coated with the HA^nano^ Surface (Promimic, Göteborg, Sweden (test)) whereas the remaining 24 implants were left uncoated (control). The coating solution was prepared according to patent US8,206,813 [47]. The coating solution consisted of 15 g water, nanocrystalline hydroxyapatite, 70 g of poly(ethylene oxide)-block-poly(propylene oxide)-block-poly(ethylene oxide) copolymer (Pluronic L64,(EO)13(PO)30(EO)13) and 205 g of p-xylene. The HA^nano^ surface was applied by adding 50 µL of the coating solution followed by rotation at 2700 rpm for 5 s. After the coating procedure, the substrates were heated (325 °C for 5 min.) in an oxygen atmosphere to burn off the surfactants and to adhere the HA crystals on the PEEK substrate. The coating solution consisted of nanosized hydroxyapatite crystals (length 20–50 nm and width 2–10 nm) which were dispersed with surfactants, water and organic solvent, as previously described [27].

### 4.3. Coating Adhesion

Sawbone 40 PCF was used as a model system to simulate the mechanical wear created by insertion. The insertion and removal torque of the implants was monitored with a Tohnichi BTG90CN torque gauge (Tohnichi America Corp, Buffalo Grove, IL, USA). Three coated and three uncoated implants were used for the measurement. Scanning electron microscopy (SEM) analysis revealed that residues of the Sawbone material remained on the implant surface after removal, these residues were removed by sonicating each implant in 15 mL ethylene diamine (purum, ≥99.0%, Fluka) for 15 min, using an ultrasonic cleaner (VWR USC100T, VWR, Leuven, Belgium). The implants were analyzed with a scanning electron microscope (SEM, LEO Ultra 55, Zeiss, Oberkochen, Germany) prior to and after the insertion testing.

### 4.4. Morphological Characterization

The surface morphology was evaluated using SEM (LEO Ultra 55 FEG, Zeiss, Oberkochen, Germany) at an accelerating voltage of 2–4 kV. To make the surface conductive, the implants were sputtered with gold in a Jeol JFC-1100E ion sputter (JEOL Ltd., Tokyo, Japan) at 10 mA for 90 s. The nano- and microtopography was described in a previous report [27].

### 4.5. Surgical Procedure and Implantation

**Figure 9:**
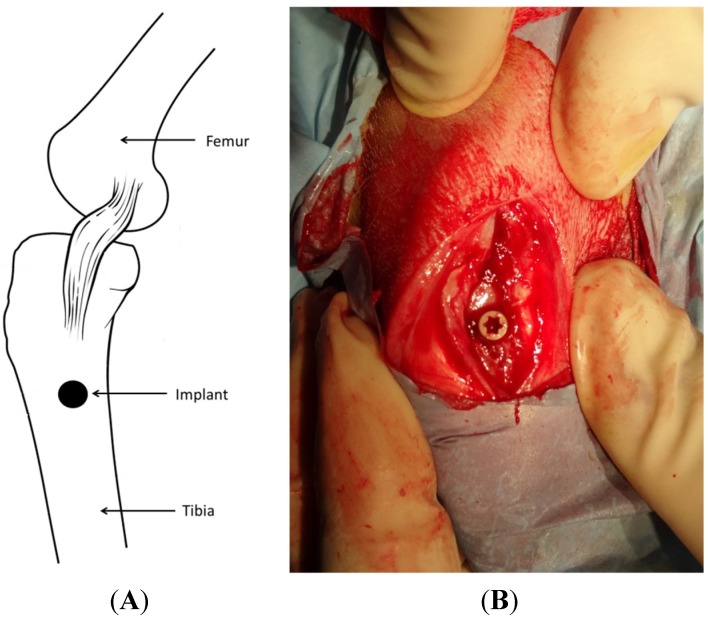
Illustration of implant location (**A**) and exposed surgical site with implant in position (**B**).

This experimental study was approved by Malmö/Lund, Sweden, Regional Animal Ethics Committee. Twenty-four lop-eared rabbits with mixed gender and an approximate mean weight of 4.1 kg (Range: 3.4–5.1 kg) were used for the study. The rabbits were divided into two groups, each consisting of 12 animals, for observation periods of either 3 or 12 weeks after implant surgery. Three weeks refers to the early stage of new bone formation which continues until week 12 where complete bone healing has occurred. The animals were administered a dose of 0.15 mL/kg medetomidine (1 mg/mL Dormitor; Orion Pharma, Sollentuna, Sweden) and 0.35 mL/kg ketamine hydrochloride (50 mg/mL Ketalar; Pfizer AB, Sollentuna, Sweden). After removing the fur, the skin of the intended surgical sites (Figure 9) was disinfected with 70% ethanol (Solveco AB, Rosersberg, Sweden) followed by 5 mg/mL chlorhexidine (Fresenius Kabi AB, Uppsala, Sweden). Local anesthesia with lidocaine hydrochloride (Xylocain, AstraZeneca AB, Södertälje, Sweden) was injected subcutaneously (approx. 1 mL) to reduce the amount of blood at the surgical site. A skin incision was made over the tibia crest inferior to the joint. The tibia bone was exposed by separating the fascia and muscles. The implant sites were prepared with a series of drills up to a final diameter of 3.2 mm. Tapping and insertion was performed manually by the operator. Each animal received two implants, one of each group, with randomized placement. Prior to suturing, the wound was cleaned and generously irrigated with sterile saline. The incisions through the fascia and skin were closed separately with bioresorbable sutures (Ethicon, Norderstedt, Germany). The animals were administered with post-surgical analgesic buprenorphine hydrochloride (0.5 mL Temgesic; Reckitt Benckiser, Slough, UK) for 3 days and were kept in normal cages until euthanasia. Euthanasia was performed with an overdose of sodium pentobarbital (60 mg/mL, Apoteksbolaget AB, Stockholm, Sweden). The bone-implant blocks were retrieved *en bloc* and immersed in 4% buffered formaldehyde for 24 h for fixation purpose.

### 4.6. Micro-CT Evaluation

The samples were increasingly dehydrated in graded ethanol up to 99.9% (Solveco AB, Rosersberg, Sweden). Ethanol was cleared gradually and the tissue simultaneously infiltrated with resin without decalcification and finally embedded in light curing resin (Technovit 7200 VLC; Heraeus Kulzer Wehrheim, Germany).

The embedded specimens were scanned in air with a microfocus X-ray CT (MCT-CB100MF Hitachi, Medico, Tokyo, Japan). Prior to scanning, the sample was pre-scanned and placed in a custom jig and aligned in an axial direction vertical to the long axis of the implant. The sample was imaged with 201 µCT slices with a single slice resolution of 51 µm. X-ray energy level was set to 70 kV and a current of 100 µA and scanned for 120 s. The threshold was calculated by discriminant function analysis, around 2200 voxel value.

The raw data from the micro-CT were reconstructed into DICOM format, imported and evaluated in a volume analysis program. (TRI 3D-BON, Ratoc system engineering, Japan) In the software the implant was vertically aligned and observed in an axial top view. To define the VOI, the implant midpoint was carefully marked out and the diameter of the implant except the threads was excluded. The slice of the implant top was selected and the length of the implant was included to VOI. The VOI was divided into three adjacent radius volumes with the same length as the implant (Figure 3). The inner (D1) region was selected to have the same thickness as the thread depth (64 voxels = 3.26 mm) and the outer two regions (D2, D3) were set to the same thickness as D1. In order to quantify the bone morphometry, the structural properties of cortical and trabecular bone near the implant were assessed with the following parameters; bone volume to total volume ratio (BV/TV, %), bone surface per given bone volume (BS/BV, %), trabecular thickness (µm), trabecular number (mm^−1^) and trabecular separation (µm). BV/TV indicates the fraction of the selected VOI that is occupied by mineralized bone. BS/BV reflects the bone surface on the trabecular bone in the VOI and can in bone biology provide a value of trabecular bone lining cells for potential osseointegration.

### 4.7. Histomorphometry

Embedded samples were cut and ground into sections with a thickness of approximately 20 µm using a diamond blade and grinding system (Exakt, Apparatebau, Norderstedt, Germany). The sectioned samples were stained with Masson-Trichrome-Goldner stain, which has three color staining suitable for distinguishing cells and bone from connective tissue (Donath and Breuner 1982). Due to the inertness of PEEK the current staining protocol was selected with the intention to reveal non-mineralized and connective tissue in the implant-bone junction (Noiset 1999). The samples were examined under light microscopy (Eclipse ME600, Nikon, Tokyo, Japan). The percentage of bone-implant contact (BIC) and new bone area (BA) were calculated with image analysis software (Image J v. 1.43r, National Institutes of Health, Bethesda, MD, USA).

### 4.8. Statistics

Statistical analysis was performed using SPSS software (Version 20, SPSS Inc., Chicago, IL, USA). The results from the histomorphometric and micro-CT analysis were subjected to a non-parametric Wilcoxon-signed rank test (Exact sign, 2-talied). P-values < 0.05 were considered significant.

## 5. Conclusions

This study reported a significant improvement of early bone integration for PEEK implants coated with nanosized HA. The results may be of clinical interest for early loading applications, but further studies are required to statistically verify the results and to improve the extended effect of the coating.
